# Exploring how medical students learn with the help of a digital presentation: a qualitative study

**DOI:** 10.1186/s12909-019-1569-z

**Published:** 2019-06-13

**Authors:** Mary Hyll, Robert Schvarcz, Katri Manninen

**Affiliations:** 1 Department of Medicine Huddinge, Karolinska Institutet, Huddinge, 141 86 Stockholm, Sweden; 2 Department of Infectious Diseases I63, Karolinska University Hospital, Huddinge, 141 86 Stockholm, Sweden; 3 Department of Infectious Diseases I73, Karolinska University Hospital, Huddinge, 141 86 Stockholm, Sweden

**Keywords:** Medical student, Medical education, E-learning, Blended learning, Presentation software, Qualitative content analysis

## Abstract

**Background:**

The web-based presentation software Prezi was used to create a digital presentation in order to facilitate antibiotic knowledge in an undergraduate course on infectious diseases in the Karolinska Institutet Medical Programme. It was unclear how the students used this in their learning, and there is a lack of research on using Prezi presentations in higher education, as well as on learner-content interaction in blended learning in general.

**Methods:**

A qualitative study design was used for an in-depth exploration of the students’ experiences of using the presentation in their studies. Students were interviewed using a semi-structured interview guide. The interviews were transcribed verbatim and analysed using qualitative content analysis.

**Results:**

Two main themes emerged from the analysis. Firstly, the students experienced that they *own their learning*: the presentation provided flexibility in studying and increased engagement in the learning process. Secondly, the presentation was part of a *superficial learning process*: students saw it as a complement to other educational activities, but expressed that there was an absence of pedagogical encounters which prevented the information in the presentation to be placed in a larger context.

**Conclusions:**

The Prezi presentation when used as an e-learning tool was a useful part of and a complement to blended learning in medical education but cannot replace face-to-face learning situations, especially not when the content of the course is complex, such as in the case of antibiotics. The learning objectives should be connected to a learning theory and made explicit for the students. Students should receive instructions and support during the course on how to use new e-learning tools. Continuous pedagogical interaction with feedback and reflection between students, teachers, and patients should be provided to enhance deep learning.

## Background

E-learning is ubiquitous in medical education and has the potential to enhance learning since students attain deeper learning when combining both words and pictures rather than words alone [1–3]. However, learning is a complicated, multidimensional process. Illeris [4] describes three dimensions of learning and competence where learners actively construct their learning within an integration of both internal and external processes. One cannot only regard a learner’s cognitive processes but must also take into consideration the emotional and social dimensions. Illeris’ model can be placed within constructivist approaches to learning, an approach which we share, and which is grounded in the belief that each student actively creates their learning in a unique way, building upon previous knowledge. E-learning, defined as “Learning conducted via electronic media, typically on the Internet” [5], and the various digital tools used in e-learning can be designed and analysed within the scope of constructivism [3].

Blended learning is a part of e-learning and is defined as a combination of face-to-face learning and asynchronous or synchronous learning using digital tools [6, 7]. Implications for using blended learning are many: transcending space and time boundaries, improving individualised and collaborative learning, the possibility of reusing learning activities, and providing updated information. In medical education, blended learning has become widely used due to its synthesising both traditional and e-learning [6].

The Medical Programme at Karolinska Institutet (KI) in Sweden is no exception in using blended learning within its curriculum. The university’s online Learning Management System (LMS) is the repository for course information, and blended learning is used in several courses, with digital material such as virtual patients, online quizzes, and multimedia presentations.

The Medical Programme at KI includes a three-week course on infectious diseases in which one of the learning objectives is the knowledge of antibiotics and how to use them in clinical practice. Traditionally, this has been taught through literature, lectures, seminars, and clinical rounds.

SOLO (Structure of the Observed Learning Outcome) taxonomy [8], used in the course’s definition of intended learning outcomes, describes learning in several levels, from the lowest prestructural learning to the highest extended abstract level. To pass the course, students should have a relational knowledge of diseases and treatment, which implies being able to compare/contrast, explain causes, analyse, relate and apply their knowledge. Not only do the students need to learn about diseases and treatment, but in that context, they must become fluent in the complicated nomenclature of antibiotics. Memorizing the many names of antibiotics can be seen as an example of *mechanical learning* [4], with the words having little context for students new to the area, which causes confusion and creates a barrier to learning. This confusion is reflected in the results of end-of-course student evaluations which have shown that antibiotic knowledge is considered to be one of the most difficult aspects of the curriculum.

In 2013 the course leaders changed the course curriculum in order to facilitate the learning of antibiotics. Classroom lectures on antibiotics were expanded, new handouts and brochures were distributed, and e-learning in the form of online quizzes as well as a digital presentation were added to the course’s webpage. The presentation was made using the web-based presentation software Prezi [9]. Prezi enables the creation of “zoomable” presentations on a desktop canvas, similar to a chalkboard, where the entire presentation can be accessed in a linear or non-linear fashion, in contrast to slide-based programs such as PowerPoint. The presentation was available to watch via the LMS, via the Prezi company’s homepage, or via the Prezi mobile app, which at the time only worked on Apple devices. Prezi was chosen since it would be free-of-charge for the university, it included animation and audio functions, and could be easily edited in the future if necessary. The canvas background and zooming possibilities were intriguing since they differed from traditional animated PowerPoint presentations by enabling the presentation to be seen in a non-linear pattern, with quick access to all slides. The course leaders thought that these features might be more useful than a traditional linear presentation. The presentation was intended to provide a basic orientation in common bacteria, related diseases, and antibiotics. It was based on a course leader’s schematic explanation of which antibiotics are effective against which type of bacteria. The presentation was divided into three main parts delineated by circles: one part on common bacteria, one on different groups of antibiotics, and one part called the “antibiotic tree”. These were placed on a canvas with the background of an orienteer running through a forest scene. The entire presentation translated to English is available online [10].

Two questions on the subject of antibiotics were included in end-of-course evaluations before the pedagogical changes were made, as well as after the changes, to assess student opinions. The students were positive to the changes, but it was difficult to relate their answers to any specific parts of the new curriculum.

The evidence strongly suggests that blended learning is as effective as or superior to traditional instruction [6, 11, 12]. However, two studies regarding blended learning research [13, 14] found a lack of attention to learner-content interaction and there is little evidence that can assist teachers in choosing the most effective approaches [15]. A recent review [16] concluded that most studies explored LMS data (log data, clicks and time used for online resources) resulting in outcomes in terms of patterns of usage. To our knowledge, only a small amount of research has been done on Prezi presentations used for facilitating learning in higher education. Virtanen et al. [17] found that students were generally positive when Prezi was introduced as an educational tool and that it can facilitate different learning styles. Casteleyn et al. [18] created two identical online lectures, one using Prezi and the other with PowerPoint. They found no difference in cognitive load, self-efficacy or knowledge gain, but that Prezi was preferred by the students.

Based on the results of our own student evaluations, the lack of research on Prezi when used as an e-learning tool, and on learner-content interaction in blended learning in general, we found it of great interest to examine in more detail how medical students used the presentation in the context of learning about antibiotics.

## Methods

### Aim

This study aimed to explore the students’ learning experiences while using the Prezi presentation. What were the advantages or disadvantages of using this software as an e-learning tool? How did the students interact with it and use it to support their learning?

### Design

A qualitative study design was used. Qualitative content analysis was chosen since this method allows the deep exploration of experience, as well as interpretation of the data, leading to conclusions about the meaning of these experiences.

### Setting

The study was held at the Department of Infectious Diseases at Karolinska University Hospital, a teaching hospital in Huddinge, Stockholm County, Sweden. The department is responsible for organising the course on infectious diseases for third-year medical students. The Medical Programme consists of 5.5 years of study, a total of 11 terms. A short course on infectious diseases is given for two days during term 5, and a three-week course is held during term 6.

### Participants

All students attending the course in the spring term of 2015 were invited to participate in the study (*n* = 78). The exclusion criterion was students who had not viewed the Prezi presentation. Information was emailed and was also presented to the students in a classroom setting by one of the authors (MH), who did not participate in teaching activities, but had collaborated on developing the presentation and had previously met the students in the role of course administrator.

A pilot interview was conducted with one student in the fall term of 2014, and this interview was included in the study. Fourteen students chose to participate and were included after first being given oral and written information and giving informed consent. Including the pilot interviewee, 8 participants were female and 7 male, between the ages of 21 to 35 years (mean age 25.3 years).

### Data collection

Semi-structured interviews with an interview guide (Table 1) were used to collect the data [19]. The guide allowed the collection of data in a flexible manner: follow-up questions were asked and new areas probed so that other topics could emerge during the interview process.

**Table 1:** Semi-structured interview guide

1. Can you tell me how it has been for you to study antibiotics in general during the course?	
2. The course offers different educational tools – digital, traditional – what are your impressions of the different kinds?	
3. How have you used the Prezi presentation in your studies?	
4. What has been the worst thing about the Prezi presentation?	
5. What has been the best thing about the Prezi presentation?	
6. How could the Prezi presentation be improved?	
7. Is there anything else you would like to say?	

The students were interviewed individually or in groups of 2–3 (8 interviews total, including the pilot), by MH. The interviews were digitally recorded. During one group interview, the interviewer noted that it was unclear if one participant had seen the antibiotic presentation or another Prezi used in the course. It became clear after transcribing the interview and reviewing the interview text in its entirety that the student had seen the correct presentation. The number of students and length of the interviews are presented in Table 2.

**Table 2:** Overview of the interviews

Interview number	Date	Number of students	Length
1 (pilot)	Dec 2014	1	25 min.
2	Feb 2015	1	29 min.
3	Feb 2015	2	34 min.
4	Feb 2015	3	61 min.
5	Mar 2015	1	24 min.
6	May 2015	3	30 min.
7	May 2015	2	54 min.
8	May 2015	2	28 min.

### Data analysis

Qualitative content analysis is a systematic approach based on analysing and interpreting text, providing a deeper meaning of the data [20]. Data in the form of text is systematically coded so that patterns and themes can be identified. This is the manifest content of the data. The deeper meaning of these themes is then interpreted, which is referred to as the latent content [21].

The analysis of data, presented stepwise, was conducted by MH and KM, and discussed with RS.

1. The recordings were transcribed verbatim by the interviewer (MH) onto Word files, one file per interview. Each line in the text was numbered to later simplify identifying areas of text.
2. The interviews were printed and read through several times by MH.
3. Text in each interview was colour-coded according to which question it had addressed. (On question 6, regarding how the presentation could be improved, comments addressing how the course itself could be improved were included).
4. Each colour-coded section from each of the interviews was cut and pasted into new Word files, so that every new file contained text that pertained to only one specific area. In the case that text could be considered to address different questions at the same time, then that text was copied into several files.
5. The text was condensed: each section of text which addressed one subject was described concisely.
6. The condensed text was coded: a few words were used as a label to describe it.
7. The codes were sorted, resulting in seven lists of key words, each relating to one question area in the interview guide. This is the manifest content of the text. The sorting and all following procedures in the data analysis were performed by the interviewer (MH) and the supervisor (KM) in collaboration.
8. Codes were reviewed and grouped into sub-categories.
9. Sub-categories were sorted and abstracted into four main categories. This process was guided by the research questions.
10. The categories were interpreted and constructed into four sub-themes and two main themes. The interpretation aimed to describe the underlying meaning of the main categories related to learning, the latent content of the text.

## Results

The content analysis resulted in two main themes: the students experienced that they *owned their learning* while using the presentation, and that the presentation was part of a *superficial learning process*. Within the first theme, regarding ownership of learning, there were sub-themes of *the e-learning tool as a support*, and *interacting with technology*. Within the theme of superficial learning, the sub-themes were that the presentation was a *complement* to other educational tools, and that the students experienced *an absence of pedagogical encounters* with teachers and patients, which impeded a deeper learning (Table 3).

**Table 3:**
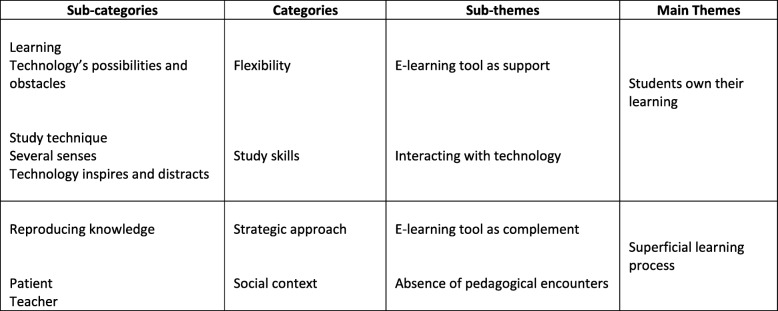
Radiographic examination of BH (changes in bone height surrounding the implant)

### Students own their learning

When offered the online, multimedia presentation as a pedagogical support, students experienced flexibility and an increased engagement in their learning.

#### E-learning tool as support

The presentation was experienced as a support in the learning process, providing freedom of choice in how to study and learn, and this was reflected in the flexibility of how the students used it. They appreciated that it could be watched where, when, and in the pace they wished. This flexibility gave them time to reflect, rewind, take notes, repeat, and not lose focus. Students watched the presentation on both home and school computers and also while traveling, on mobile devices that supported the Prezi app. The students expressed positive associations with electronic devices and experienced watching the presentation as a relaxing activity, since they could choose to watch it when they were in the “right” mood, in contrast to traditional lectures, when they had no control over when they had to attend. If something was unclear, they searched for answers online and thus avoided having to ask “embarrassing” questions.

One student expressed the experience of using a digital presentation as support as follows:

*“Large parts of a student’s everyday life are spent in front of some kind of electronics, and that makes you feel more comfortable with it. It must be something psychological, but I think that the “have to” disappears: that I “have to listen” or “have to study”, “have to be serious”. The pressure disappears and when you’re relaxed it’s easier to absorb things.” Student 3, group interview*

However, students experienced technical obstacles as well. They did not always succeed in viewing it on their chosen device. Sometimes it did not load, or loaded very slowly. The majority of the students had not previously used Prezi, and some did not understand that it could be paused, “rewound” and that there was a zooming function. The pausing, in particular, was frustrating, since it often caused an entire section to be replayed, and there was no way to repeat small sections of a sound file without the file starting over from the beginning. In these cases the technology did not support their learning but hindered it.

#### Interacting with technology

Many students interacted with the presentation by watching it in a linear manner, due to not understanding the technology, and thus its canvas format was not used. Some students did not understand the autoplay function, and instead clicked through the presentation, one section at a time, which they described as a positive influence on their engagement.

While watching it, they transcribed the presentation, or summarized it with key words. They watched and listened, paused, transcribed, and repeated unclear sections. One section called “The antibiotic tree” was mainly used as a lexicon of sorts, and several students did not review it. Some students made screen shots of different sections, which they printed out.

Students experienced the multimedia presentation as better than reading only text, and described that its strength was that it engaged several senses. By seeing colourful images, the students’ visualization became easier. However, the visual structure of the information was not always evident to the students, which caused confusion.

Interacting with the technology was described as stimulating learning in some cases, and in others distracting. When the technology worked smoothly, it increased interest in learning. The students appreciated being able to pause and some tested the zooming function. The layout was helpful to some and distracting to others. The sound, in particular, was experienced as both helpful and distracting. It caused confusion for some students when they did not see the words on the screen matching those being read aloud.

The following quote illustrates how one student interacted with the technology:

*“I watched the Prezi presentation from start to finish to get an idea what it was. I listened, paused, took notes, listened, paused, took notes, and when I thought I had most of it down on paper, I listened again to make sure that I had everything, that what I had written was correct. In that way you get the information through sound, through sight and by writing yourself, and then it feels like you have absorbed it through all possible ways of learning.” Student 1, individual interview*

### Superficial learning process

The students experienced the presentation as giving an overview, helping them to orient themselves within a complicated subject. It was a complement to other pedagogy and provided guidance. The students expressed that there was a lack of interaction with teachers and patients, which they felt was necessary for placing the knowledge in a context.

#### E-learning tool as complement

*“It is a complement because we do have a lecture on antibiotics. The point of the Prezi presentation is that you can watch it many times, have an overview and have a tool for practice.” Student 2, individual interview*

The students saw the presentation as a complement to other pedagogical activities offered in the course. Some students used the Prezi presentation as a base for guiding their studies, and built upon its structure. They sorted and categorized antibiotics, and expanded their knowledge using other sources. Some experienced it as containing too much information, with a complicated diagram, and thus relied more on a handout with antibiotics in table form to assist them with orientation and visualization of different antibiotic categories. Others experienced that the presentation helped simplify the subject, but the context was missing. They also expressed that the learning objectives for the course were unclear or missing. They described a lack of more nuanced, in-depth knowledge about antibiotics.

#### Absence of pedagogical encounters

*“There is something missing, you know, when there isn’t a real person to answer questions, like there is at a lecture.” Student 13, group interview*

There was no follow-up discussion in connection with the presentation, and this lack of feedback was also described as hindering deeper learning. The teacher’s voice was featured on the presentation, with no video of him, and students expressed a preference for seeing the teacher, remarking that it is easier to remember information when one sees body language.

The students expressed that knowledge must be formed while having patient contact and would have liked to have had more clinical practice. Connecting knowledge to patients enabled them to put the information in a larger perspective, giving it meaning.

*“At the ward I learn so much because I can see things for myself. For example I have been terrible at antibiotics, but when you can connect them to something, when you remember which one they gave to which patient, then the knowledge sticks…” Student 7, group interview*

## Discussion

The aim of the study was to explore students’ learning experiences while using a Prezi presentation in the context of learning antibiotics. It had been created with the hope of facilitating study, to help the students orient themselves in this complicated subject, to perhaps be more useful and enjoyable than, for example, written information on paper, since colourful pictures and audio could be beneficial.

Interestingly, the results of the study are somewhat contradictory. On one hand the students clearly appreciated the presentation, which enabled them to take ownership of their learning, but on the other hand learning seemed to remain a superficial process. So how can this contradiction be understood or explained? Our suggestion is that although the presentation enhanced student engagement and involvement, the learning did not occur on deeper lever due to absence of social interaction and lack of explicit learning objectives that are related to learning theory.

The results showed that students had a positive attitude to the presentation and were engaged in their learning. The presentation was described as useful and supportive for learning. Usefulness and support were based on experiencing it as flexible, user-friendly and easy to access, meeting their learning needs and empowering them during their studies. This aligns well with results from other studies [22–25].

However, technical problems can distract and cause obstacles. Some students had technical difficulties with the presentation and trouble understanding its different functions, which surprised us. The fact that the presentation could be used in a non-linear manner did not seem to be a pedagogically advantageous aspect, with some students expressing that the technical problems became such a barrier that they were disinterested in viewing the presentation more than once, and instead turned to information that was easier to access, for example YouTube videos or paper handouts. The majority of the students in our study were born in the 1990s and had grown up with the internet. We assumed that using a digital program would not present any problems. Our results are similar to Duffy et al. [26], who found that the main problems of Prezi presentations were of a technical nature and that students had difficulty understanding how to use the software. In a 2018 review of barriers and solutions to online learning in medical education [27] O’Doherty et al. found that one of the main barriers was the lack of technical skills in educators. As novices in e-learning, we were not familiar with the concept of usability testing [28]. Had we tested the technology with just a few users, we would have most likely discovered the difficulties and could have prevented at least some of them from becoming a barrier. After the completion of the study, some of the technical problems were solved by informing students about using a different web browser as well as explaining the functions of the Prezi software.

Accordingly, students’ experiences of owning their learning can be related to the concept of usability. Asarbakhsh and Sandars [28] mean that usability in relation to e-learning can be defined as the ability to use and to gain knowledge from learning technologies with ease and satisfaction. Further, usability is also about knowing the learner and the context, technological aspects, and that the content is consistent with the learning objectives [28]. Students in our study expressed that there should be clear learning objectives. However, they did not always experience that this was the case in this course, which may have affected the overall experience of usability. The concept of usability also includes the connection to learning theories. Sandars et al. [29] and Masters et al. [2] stress the importance of underpinning theory and making it explicit, which provides insight into how to facilitate students’ learning by using technology.

Even though the flexibility of the presentation was experienced as positive, it lacked a place within a larger context, and was thus challenging to use for deep learning. Instead, it became part of a fragmented approach to learning, where meaning-making was difficult to achieve. Meaningful learning consists of students seeking to make sense of their experiences in an active cognitive process that requires more than recalling or recognizing facts [30]. The presentation temporarily engaged the students by appealing to their interest in digital technology, but it contained abstract and complicated information that did not include an emotional or social dimension [4]. The presentation helped the students acquire an overview of and an orientation within a complicated subject: antibiotics. Thus, students’ learning did not reach a deep level but remained superficial. A possible explanation could be the lack of interaction with teachers, patients and with peers. More interaction with patients could have enhanced the students’ understanding of antibiotics related to the diagnosis and relevant management. Patients could also have contributed to students’ learning by giving insights to their situation as a whole.

Interaction with teachers and peers, either face-to-face, or via digital technology, could have enhanced the students’ understanding of the theoretical knowledge of antibiotics and clinical reasoning. Students explicitly expressed the importance of interacting with both teachers and patients, but interestingly they did not mention interaction with peer students. It has not been financially feasible to increase clinical time during the course, but technology could be used to create a more patient-centred education. Lajoie [31] presents several examples of how the real world can be explored by using video triggers, standardised patients, verbal and text chats, and virtual worlds.

The presentation could be improved by including social and emotional elements, for example embedding videos with patient cases connected to the different antibiotics, or including questions that trigger increased reflection.

Social and emotional dimensions are essential for students to place the fragments of knowledge into a larger, meaningful context, to construct an understanding in an interactive process with other people [8, 31–33]. When planning on using any tools in e-learning, it is essential to remember that this process is a pedagogical interaction and an important aspect of deep learning [34, 35].

## Conclusion

The Prezi presentation when used as an e-learning tool can be a useful part of blended learning but cannot replace face-to-face learning situations, especially not when the content of the course is complex, such as in the case of antibiotics. The learning objectives and pedagogical activities should be connected to a learning theory and made explicit for the students. Students should also receive instruction and support during the course on how to use e-learning tools to receive the most benefit of them. Continuous pedagogical interaction with feedback and reflection between students, teachers and patients should be provided to enhance deep learning.

### Limitations

This study was a small-scale study conducted within a specific course in one teaching hospital. Therefore, the context and the setting are described in detail aiming to enhance the judgement of transferability. Also, relating the results in theoretical concepts is an attempt to enhance the transferability of the results.

## Abbreviations

KI: Karolinska Institutet
KM: Katri Manninen
LMS: Learning Management System
MH: Mary Hyll
RS: Robert Schvarcz
SOLO: Structure of the Observed Learning Outcome
