# Correction to: Exploring how medical students learn with the help of a digital presentation: a qualitative study

**DOI:** 10.1186/s12909-019-1694-8

**Published:** 2019-07-08

**Authors:** Mary Hyll, Robert Schvarcz, Katri Manninen

**Affiliations:** 1 Department of Medicine Huddinge, Karolinska Institutet, Huddinge, 141 86 Stockholm, Sweden; 2 Department of Infectious Diseases I63, Karolinska University Hospital, Huddinge, 141 86 Stockholm, Sweden; 3 Department of Infectious Diseases I73, Karolinska University Hospital, Huddinge, 141 86 Stockholm, Sweden

**Correction to: BMC Med Educ**

**https://doi.org/10.1186/s12909-019-1569-z**

Following publication of the original article [1], the author reported that Table 3 was given the incorrect heading.

**Incorrect heading for Table 3 in the original article:**

Radiographic examination of BH (changes in bone height surrounding the implant).

**Correct heading for Table 3:**

Overview of the sub-categories, categories, sub-themes and main themes which emerged in the study.

The original article has been corrected.
